# Personalized Care and Treatment Compliance in Chronic Conditions

**DOI:** 10.3390/jpm12050737

**Published:** 2022-05-01

**Authors:** Júlio Belo Fernandes, Fábio Teixeira, Catarina Godinho

**Affiliations:** 1 Escola Superior de Saúde Egas Moniz, 2829-511 Almada, Portugal; cgodinho@egasmoniz.edu.pt; 2 Grupo de Patologia Médica, Nutrição e Exercício Clínico (PaMNEC), Centro de Investigação Interdisciplinar Egas Moniz (CiiEM), 2829-511 Almada, Portugal; 3 Life and Health Sciences Research Institute (ICVS), School of Medicine, University of Minho, 4710-057 Braga, Portugal; fabiogrodri@gmail.com; 4 ICVS/3B’s Associate Lab, PT Government Associated Laboratory, 4710-057 Braga, Portugal; 5 Medical and Industrial Biotechnology Laboratory (LABMI), Porto Research, Technology, and Innovation Center (PORTIC), Porto Polytechnic Institute, 4200-465 Porto, Portugal; 6 I3S—Instituto de Investigação e Inovação em Saúde, Universidade do Porto, 4200-135 Porto, Portugal

Chronic diseases are commonly defined as conditions that last one year or more and require ongoing medical attention, limit activities of daily living, or both [1]. These include, for example, diabetes, cancer, and cardiovascular and neurodegenerative disorders. Such diseases are growing at an alarming rate, especially in the aging population, and are the leading causes of death and disability for adults in developed countries [2].

Living with a chronic condition can be stressful because it changes patients’ lives, distressing their physical or/and mental health or threatening their survival [3]. Nevertheless, people are able to take steps to cope with these new situations, manage their condition, and maintain a good quality of life.

People who have chronic diseases spend a significant amount of time in self-management in out-of-hospital environments, in their homes, and in their community settings. These patients have different disease statuses and management requirements.

Medicine and healthcare have been profoundly transformed as a result of technological progress along with clinical research achievements, resulting in an increased disease-management capacity [4].

Over time, medicine and healthcare models have evolved towards a practice that is technically feasible, economically valuable [4,5], and culturally, ethically, and socially accepted. In this evolution, personalized care could be the key. It represents an opportunity to improve care for all individuals from a singular or collective point of view that holds promise for the prevention and treatment of diseases.

The development of personalized care implies strong involvement and commitment from society. Researchers and policymakers must analyze the potential effect of personalized care approaches within healthcare and recommend reorganization of services, infrastructures, regulations, and policies for personalized care to become truly embedded/implemented in healthcare systems [4]. Additionally, patients, caregivers, family, and healthcare providers identify and discuss problems caused by or related to the patient’s condition and then develop plans and goals to empower patients and their families.

A personalized care approach could greatly benefit patients with chronic conditions given its impact on aspects of physical health, mental health, and the ability to self-manage conditions. New approaches that allow for the development of personalized care and improvement of overall treatment adherence should be strongly encouraged by healthcare providers.

With this editorial and Special Issue focusing on personalized care and treatment compliance in chronic conditions, we aimed to stimulate the research community to continue producing evidence that supports the positive effects of a personalized care approach on the diagnosis and treatment of chronically ill patients.

The accepted topics included a variety of research studies, from study protocols [6] to original qualitative [7,8,9] and quantitative studies [10,11,12,13,14,15,16,17]. Additionally, review protocols [18,19] and literature reviews [20,21,22,23] were included, covering different areas of care, from studies addressing ageism [6] or stigma [17] to a personalized approach to diagnosing and managing ischemic stroke [16]. We highlighted the importance of recognizing that in order to enhance healthcare delivery, all stakeholders involved in providing care must actively partner with patients and families to enable changes in the care process, obtaining higher patient satisfaction and better health outcomes. Therefore, studies involving family caregivers, which are central to delivering better healthcare, were also considered and published in the Special Issue.

Several internationally renowned research groups contributed with research. We highlight two studies in particular. The first study reported on a novel boot-camp program to help guide personalized exercise in people with Parkinson’s disease. This study shows the program’s acceptability and usefulness regarding participation in a PD-personalized educational and exercise boot-camp program. This program was considered a valuable example of personalized care used to better influence patients’ exercise habits [15]. Second, we highlight a study aiming to assess the effect of virtual-reality-based therapy to reduce the impact of fibromyalgia syndrome in outcomes such as pain, dynamic balance, aerobic capacity, fatigue, quality of life, anxiety, and depression. The findings demonstrated that virtual-reality-based therapy can effectively reduce pain, fatigue, anxiety, and depression and increase dynamic balance, aerobic capacity, and quality of life in women with fibromyalgia syndrome [21].

Overall, 19 articles were published, all with a common aim of increasing knowledge in the field of personalized care.
